# Octyl Gallate Markedly Promotes Anti-Amyloidogenic Processing of APP through Estrogen Receptor-Mediated ADAM10 Activation

**DOI:** 10.1371/journal.pone.0071913

**Published:** 2013-08-15

**Authors:** She-Qing Zhang, Darrell Sawmiller, Song Li, Kavon Rezai-Zadeh, Huayan Hou, Shufeng Zhou, Douglas Shytle, Brian Giunta, Frank Fernandez, Takashi Mori, Jun Tan

**Affiliations:** 1 Rashid Laboratory for Developmental Neurobiology, Silver Child Development Center, Morsani College of Medicine, University of South Florida, Tampa, Florida, United States of America; 2 Department of Neurology, Shanghai Changhai Hospital, Shanghai, China; 3 James A. Haley Veteran’s Administration Hospital, Tampa, Florida, United States of America; 4 Department of Biophysics, School of Physics and Optoelectronic Technology, Dalian University of Technology, Dalian, China; 5 Department of Therapeutic Science, School of Pharmacy, University of South Florida, Tampa, Florida, United States of America; 6 Center of Excellence for Aging and Brain Repair, Department of Neurosurgery, Morsani College of Medicine, University of South Florida, Tampa, Florida, United States of America; 7 Neuroimmunology Laboratory, Department of Psychiatry and Behavioral Neurosciences, Morsani College of Medicine, University of South Florida, Tampa, Florida, United States of America; 8 Departments of Biomedical Sciences and Pathology, Saitama Medical Center and University, Kawagoe, Saitama, Japan; Torrey Pines Institute for Molecular Studies, United States of America

## Abstract

Our previous studies showed that the green tea-derived polyphenolic compound (−)-epigallocatechin-3 gallate (EGCG) reduces amyloid-β (Aβ) production in both neuronal and mouse Alzheimer’s disease (AD) models in concert with activation of estrogen receptor-α/phosphatidylinositide 3-kinase/protein kinase B (ERα/PI3K/Akt) signaling and anti-amyloidogenic amyloid precursor protein (APP) α-secretase (a disintegrin and metallopeptidase domain-10, ADAM10) processing. Since the gallate moiety in EGCG may correspond to the 7α position of estrogen, thereby facilitating ER binding, we extensively screened the effect of other gallate containing phenolic compounds on APP anti-amyloidogenic processing. Octyl gallate (OG; 10 µM), drastically decreased Aβ generation, in concert with increased APP α-proteolysis, in murine neuron-like cells transfected with human wild-type APP or “Swedish” mutant APP. OG markedly increased production of the neuroprotective amino-terminal APP cleavage product, soluble APP-α (sAPPα). In accord with our previous study, these cleavage events were associated with increased ADAM10 maturation and reduced by blockade of ERα/PI3k/Akt signaling. To validate these findings *in vivo*, we treated Aβ-overproducing Tg2576 mice with OG daily for one week by intracerebroventricular injection and found decreased Aβ levels associated with increased sAPPα. These data indicate that OG increases anti-amyloidogenic APP α-secretase processing by activation of ERα/PI3k/Akt signaling and ADAM10, suggesting that this compound may be an effective treatment for AD.

## Introduction

Amyloid precursor protein (APP) proteolysis is fundamental for production of amyloid-β (Aβ) peptides implicated in Alzheimer’s disease (AD) pathology [1]–[4]. APP proteolytic products arise from the actions of α-, β-, and γ-secretases. In the amyloidogenic pathway, Aβ peptides are produced *via* initial action of β-secretase (BACE) cleavage, which creates an Aβ-containing carboxyl-terminal fragment, β-CTF or C99 [5], [6]. This also generates an amino-terminal, soluble APPβ (sAPPβ) fragment, which is released extracellularly. Intracellularly, β-CTF is then cleaved by a multi-protein γ-secretase complex that results in generation of the Aβ peptide and a smaller γ-CTF, also known as C57 [7]. Conversely, in the anti-amyloidogenic pathway, APP is first cleaved at the α-secretase site, by the putative α-secretase (a disintegrin and metallopeptidase domain-10, ADAM10), which results in the release of amino-terminal soluble APPα (sAPPα) and the generation of α-CTF or C83 [8].

Over the past decade, there has been intense focus on investigating the processes of APP proteolysis and Aβ production as possible targets for AD therapy [9]. Various synthetic and naturally-occurring compounds have been analyzed for their efficacy in the modulation of these pathological events. Phenolic flavonoids found in green tea are some of the naturally-occurring compounds recently achieving popularity for their therapeutic properties in the treatment of AD. One of these, (−)-epigallocatechin-3-gallate (EGCG), initially reported to have anti-carcinogenic effects [10], [11], has been shown to reduce Aβ generation together with increased ADAM10 maturation as well as sAPPα and α-CTF production in various neural cell lines [12]–[15].

Several works revealed that estrogen receptors (ER) may be important for cellular binding of EGCG and other naturally-occurring phenolic compounds, including octyl gallate (OG) and atranorin (AN, an ester of gallic acid) [16]. For EGCG, it has been suggested that its gallate group corresponds to the 7α-position of estrogen [17]. Most recently, we found that ERα binding, and subsequent activation of phosphatidylinositide 3-kinase (PI3k) and protein kinase B (Akt) signaling pathways, plays a role in EGCG-elicited APP α-secretase processing in murine neuroblastoma cells overexpressing the Swedish mutant form of APP (N2a/APPsw cells) [18]. Because of the potential importance of the gallate group in the therapeutic effects of EGCG, we investigated the effect of other gallate containing phenolic compounds, including (−)-gallocatechin (GC), methyl gallate (MG), propyl gallate (PG), butyl gallate (BG), octyl gallate (OG), and AN, as well as two phenolic compounds lacking the gallate moiety, (−)-epicatechin (EC) and catechin (C), on ADAM10 maturation and APP α-processing in these cells. We found that two of these compounds, OG and AN, are much more potent than EGCG in inducing APP α-processing and reducing Aβ_40_ and Aβ_42_ production *in vitro* and *in vivo.* Following this, we selected the most efficacious compound (OG) and then determined the role of ER mediated PI3K/Akt activation in its APP α-processing promoting effect.

## Materials and Methods

### Ethics Statement

All experiments were performed in accordance with the guidelines of the NIH, and all animal studies were approved by the University of South Florida Institutional Animal Care and Use Committee. Animals were humanely treated during the experiment, and all efforts were made to minimize animal suffering. Animals were anesthetized with sodium pentobarbital (50 mg/kg) and then euthanized by transcardial perfusion with ice-cold physiological saline containing heparin (10 units/ml).

### Reagents

Green tea-derived phenolic compounds (95% purity by HPLC), including EGCG, EC, GC, and C, and other gallate containing phenolic compounds, including MG, PG, BG, OG, and AN (an ester of gallic acid), were purchased from Sigma-Aldrich (St. Louis, MO). The selective ERα antagonist methyl-piperidino-pyrazole (MPP) and the selective ERβ antagonist 4-[2-Phenyl-5,7-bis (trifluoromethyl) pyrazolo[1,5-a] pyri midin-3-yl] phenol (PHTPP) were purchased from Tocris Bioscience (Ellisville, MO). The PI3K inhibitor, wortmannin, was obtained from Calbiochem (San Diego, CA) and the Akt inhibitor, triciribine hydrate (TCN), was obtained from Sigma-Aldrich.

### Cell Cultures

Murine neuroblastoma cells overexpressing the human wild-type APP (N2a/APPwt cells) or Swedish mutant form of APP (N2a/APPsw cells) were cultured as described previously [15]. For primary culture of cortical neuronal cells, cerebral cortices from one day-old ERα deficient or intact mice (The Jackson Laboratory, Bar Harbor, ME) were isolated under sterile conditions and kept in 75 cm^2^ flasks with complete medium for 7 days. The primary neurons were then mechanically dissociated in trypsin (0.25%) for 15 minutes at 37°C. Cells were collected after centrifugation at 1,200×*g*, re-suspended in Dulbecco’s modified Eagle’s medium supplemented with 10% fetal calf serum, 10% horse serum, uridine (33.6 µg/ml; Sigma-Aldrich), and fluorodeoxyuridine (13.6 µg/ml; Sigma-Aldrich), and then seeded in 24-well collagen-coated culture plates at 2.5×10^5^ cells per well. These cells were cultured in RPMI 1640 medium supplemented with 5% fetal calf serum, 2 mM glutamine, 100 units/ml penicillin, 0.1 µg/ml streptomycin, and 0.05 mM 2-mercaptoethanol according to previously described methods [19]. One week after the primary culture, these cells were transiently transfected with a human wild-type APP gene (APP_695_ cDNA plasmid kindly provided by Dr. Claus Pietrzik; Johannes Gutenberg University, Mainz, GE) using the Lipofectamine LTX™ Reagent kit (Life Technologies, Grand Island, NY). In brief, for each well of these cells to be transfected, 0.5 µg of APP_695_ plasmid DNA was diluted in 100 µl of Opti-MEM® I reduced serum media containing 1.5 µl of Lipofectamine LTX™ reagent and mixed gently. After 30 minute incubation at room temperature, we added 100 µl of this complex directly in each well of these cells. After 24 hour incubation at 37°C, transient transfection was verified by immunoblotting (IB) analysis using an anti-Aβ_1–16_ antibody (6E10, 1 mg/ml; Covance Research Products, Emeryville, CA) (data not shown).

### Enzyme-linked Immunosorbent Assay (ELISA)

Soluble Aβ_40_ and Aβ_42_ species were directly detected in conditioned media collected from cultured cells or brain homogenates after a 1∶4 or 1∶10 dilution, respectively, using the Aβ_40_ and Aβ_42_ ELISA kits (IBL, Minneapolis, MN) in accordance with the manufacturer’s instructions. For preparation of brain homogenates, mouse brains were isolated under sterile conditions on ice and placed in ice-cold lysis buffer (20 mM Tris, pH 7.5, 150 mM NaCl, 1 mM EDTA, 1 mM EGTA, 1% v/v Triton X-100, 2.5 mM sodium pyrophosphate, 1 mM glycerolphosphate, 1 mM Na_3_VO_4_, 1 µg/ml leupeptin, and 1 mM PMSF) as described previously [14]. Brains were then sonicated on ice for about 3 minutes, allowed to stand for 15 minutes at 4°C, and centrifuged at 18,800×g for 15 minutes.

### Immunoblotting Analysis

Cultured cells or mouse brains were lysed in ice-cold lysis buffer and an aliquot corresponding to 50 µg of total protein was electrophoretically separated using 16.5% Tris-tricine gels. Electrophoresed proteins were then transferred to polyvinylidene difluoride membranes (Bio-Rad, Richmond, CA), washed in dH_2_O, and blocked for 2 hours with ambient temperature nonfat dry milk. After blocking, membranes were hybridized for 2 h at ambient temperature with various primary antibodies. Membranes were then washed three times for 5 minutes each in dH_2_O and incubated for 1 h at ambient temperature with the appropriate horseradish peroxidase-conjugated secondary antibody (1∶1,000; Thermo Fisher Scientific, Waltham, MA). All antibodies were diluted in Tris-buffered saline (TBS; 25 mM Tris-HCl, pH 7.4, 150 mM NaCl) containing 5% (w/v) nonfat dry milk. Blots were developed using the luminol reagent (Thermo Fisher Scientific). Densitometric analysis was done as described previously using a FluorS Multiimager with Quantity One software (Bio-Rad) [20]. Antibodies used for IB analysis include a specific anti-human sAPPα antibody (2B3, 100 µg/ml; IBL, Minneapolis, MN), anti-Aβ_1–16_ antibody (6E10, 1 mg/ml; Covance Research Products), anti-Aβ_17–24_ antibody (4G8, 1 mg/ml; Covance Research Products), anti-Aβ_1–12_ antibody (BAM10, 500 µg/ml; Sigma-Aldrich), anti-ADAM10-carboxyl-terminal antibody (ADAM10, 500 µg/ml; Sigma-Aldrich), anti-ADAM10 (mature form) antibody (anti-ADAM10, 1 mg/ml, EMD Millipore), anti-human APP carboxyl-terminal antibody (500 µg/ml; pAb751/770; Merck Millipore, Darmstadt, Germany), anti-APP-carboxyl-terminal antibody 396 (pAPP396, 500 µg/ml, kindly provided by Dr. S. Gandy), and β-actin antibody (100 µg/ml; Sigma-Aldrich).

### Mice

The transgenic AD model mice (line Tg2576) were purchased from Taconic (Germantown, NY). For intracerebroventricular (i.c.v.) administration of EGCG or OG, a total of 13 female Tg2576 mice were used; five mice received EGCG or OG, and the other three mice received phosphate-buffered saline (PBS; pH 7.4). Beginning at 6 months of age, the mice were injected with EGCG, OG, (10 µg in 5 µl PBS, respectively), or PBS (5 µl) daily for one week [21]. An additional five female and five male Tg2576 mice at 6 months of age received OG, and three (2 male/1 female) mice received PBS, to determine gender differences in the response to OG. All mice were sacrificed 24 hours after the last injection for analysis of amyloidosis.

### Statistical Analysis

All data were normally distributed. Therefore, in instances of single mean comparisons, Levene’s test for equality of variances followed by t test for independent samples was used to assess significance. In instances of multiple mean comparisons, one-way analysis of variance (ANOVA) was used, followed by *post hoc* comparison using Bonferonni’s method. Levels were set at 0.05 for all analyses. The Statistical Package for the Social Sciences, release IBM 10.0.5 SPSS (IBM, Armonk, NY) was used for all data analyses.

## Results

Previous studies have shown that the green tea-derived polyphenolic compound EGCG reduces Aβ production in both neuronal and mouse AD models in concert with activation of anti-amyloidogenic APP α-processing [14], [15], [18], [22]. This activation was shown to be mediated by activation of the putative α-secretase, ADAM10 [15], as well as ERα/PI3K/Akt signaling mechanism [18]. Since the gallate moiety in EGCG may correspond to the 7α position of estrogen, thereby facilitating ER binding [17], we investigated the effect of other gallate containing phenolic compounds, including GC, MG, PG, BG, OG, and AN (an ester of gallic acid), as well as two phenolic compounds lacking the gallate moiety, EC and C, for their ability to reduce Aβ production *via* activation of ADAM10 and anti-amyloidogenic APP α-processing.

### The Gallate Moiety is Key for Reducing Aβ Generation and Increasing APP α-proteolysis

**Figure 1:**
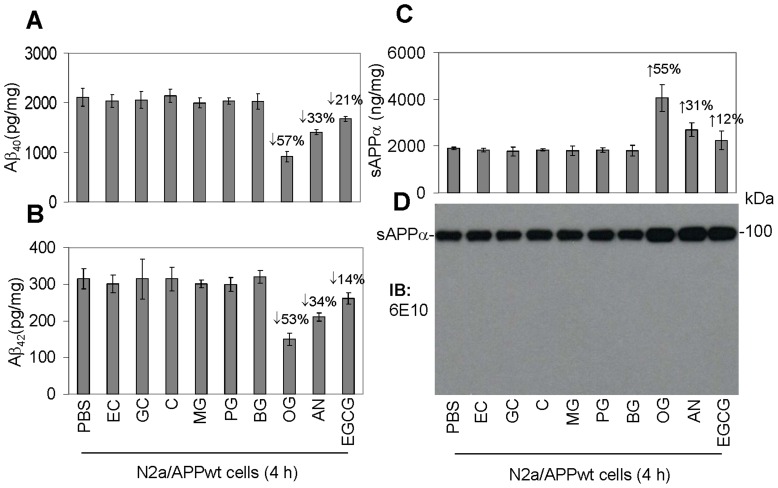
Characterization of gallate moiety in increasing amyloid precursor protein (APP) α-proteolysis and reducing amyloid-β (Aβ) generation. Given (−)-epigallocatechin-3 gallate (EGCG) significantly reduces Aβ generation from N2a/APPsw cells, we screened other gallate containing phenolic compounds, namely (−)-gallocatechin (GC), methyl gallate (MG), propyl gallate (PG), butyl gallate (BG), octyl gallate (OG), and atranorin (AN) (an ester of gallic acid) and two non-gallate containing compounds, (−)-epicatechin (EC) and (−)-catechin (C), for their ability to reduce Aβ generation (Table 1). (A-D) N2a cells overexpressing wild-type APP (N2a/APPwt) were plated in 24 well-plates (200,000 cells/well) and treated with each of these compounds at 10 µM (our previous studies indicated that this is the minimum concentration of EGCG required to noticeably inhibit Aβ production in N2a/APPsw cells) in addition to PBS control. Data are represented as pg of Aβ_40_, Aβ_42_ (A, B) or ng of sAPPα (C) in the conditioned media secreted throughout the 4 h of administration for each compound, normalized to intracellular protein (mg). sAPPα ELISA results were further supported by immunoblotting analysis (IB) using an anti-Aβ_1–17_ antibody (6E10) (D). One-way ANOVA followed by *post hoc* comparison revealed significant differences when comparing EGCG, AN and OG to each of other compounds. These results are representative of three independent experiments with three replicates for each condition. We have summarized these data in Table 1. Together, they suggest that the gallate moiety may be an important functional component in EGCG, AN and OG, consequently playing a role in reducing Aβ generation and elevating APP α-processing. We have replicated these results in cells overexpressing the Swedish mutant form of APP (N2a/APPsw cells, data not shown).

Using similar conditions as in our prior investigations [15], [18], N2a/APPsw or N2a/APPwt cells were treated with EGCG or eight other phenolic compounds at 10 µM in addition to PBS control for 4 hours. Aβ_40_ and Aβ_42_ peptides were then measured in conditioned media from these cells by ELISA (Fig. 1A, B). Similar to results from our previous studies [15], EGCG reduced Aβ_40_ and Aβ_42_ production in N2a/APPsw cells by 23 and 34%, respectively (data not shown), and reduced the production of these peptides in N2a/APPwt cells by 21 and 14%, respectively. Importantly, we found that the gallate-containing phenolic compounds, AN and OG, were much more effective than EGCG in reducing Aβ_40_ and Aβ_42_ production by these cells. Indeed, AN decreased Aβ_40_ and Aβ_42_ production in N2a/APPsw and N2a/APPwt cells by 33–37% and OG decreased Aβ_40_ and Aβ_42_ production by 53–59%, respectively. However, Aβ_40_ and Aβ_42_ productions were not reduced by any of the other phenolic compounds regardless of the presence of the gallate moiety.

Since our previous studies showed that promotion of α-secretase cleavage of APP is required for EGCG-mediated Aβ reduction [15], we determined the effect of the above listed phenolic compounds on APP α-secretase cleavage. In order to reflect the naturally-occurring APP, cultured N2a/APPwt cells were treated with EGCG or one of the other eight phenolic compounds at 10 µM, or PBS control, for 12 h and then conditioned media were collected and subjected to sAPPα ELISA and IB analyses (Fig. 1C, D). Paralleling reduced Aβ generation, EGCG, AN and OG elevated sAPPα levels in conditioned media. However, sAPPα was not increased by any of the other phenolic compounds. These data are also summarized in Table 1. Given that promotion of sAPPα production is involved in APP α-proteolysis, these results suggest that the anti-amyloidogenic effect of OG, AN, and EGCG may involve increased anti-amyloidogenic APP α-processing.

**Table 1:** Characterization of gallate moiety in increasing amyloid precursor protein (APP) α-proteolysis and reducing amyloid-β (Aβ) generation.

Structure of sAPPa activating compounds (10 mM)	R1	R2	% increasing sAPPa (mean ± SD)in N2a/APPwt cells	% inhibition of Abs (mean ± SD) in N2a/APPwt cells
(−)-epigallocatechin-3-gallate (EGCG)	OH×5	gallate	11.85% ±6.34	17.57% ±3.23
(−)-epicatechin (EC)	OH×5		0.12±0.05	3.57% ±0.23
(−)-gallocatechin (GC)	OH×3	gallate	0.09±0.02	2.40% ±0.19
(−)-catechin (C)	OH×3		0.16±0.06	−1.47% ±0.09
methyl gallate (MG)	CH_3_×1	gallate	0.06±0.01	5.37% ±2.39
propyl gallate (PG)	CH_3_×2	gallate	0.13±0.04	3.38% ±1.83
butyl gallate (BG)	CH_3_×3	gallate	0.08±0.02	3.91%±1.03
octyl gallate (OG)	CH_3_×6	gallate	54.67±0.85	55.16% ±9.82
atranorin (AN)		gallate×2	30.54±0.73	33.12% ±5.98

**Figure 2:**
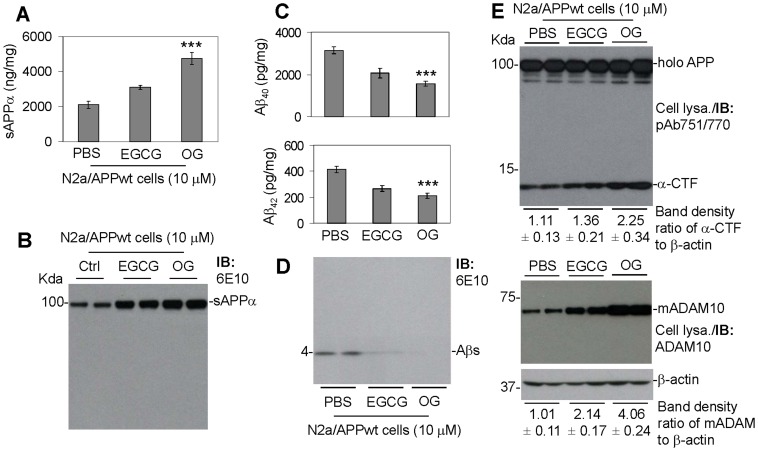
OG significantly promoted α-secretase cleavage of APP and reduced Aβ production in N2a cells overexpressing wild-type APP (N2a/APPwt cells). N2a/APPwt cells were plated in 24 well-plates (100,000 cells/well) and treated with OG or EGCG at 10 µM, a most effective dose as determined by our previous and preliminary studies, for 12 hours. The conditioned media were then collected from these cells and subjected to: (A) ELISA for sAPPα, (B) IB analyses for sAPPα, (C) ELISA for Aβ_40_ and Aβ_42_and (D) IB analysis for total Aβ species using 6E10. In addition, cell lysates (Cell lysa.) were prepared and subjected to IB analysis for carboxyl-terminal fragments of APP (CTFs) using an anti-carboxyl-terminal APP antibody (pAb751/770) (E, top) and for ADAM10 using an anti-carboxyl-terminal ADAM10 antibody (ADAM10) (E, bottom). As shown below each IB panel, densitometry analysis shows the band density ratios (mean ± SD) of α-CTF to β-actin and mature ADAM10 (mADAM10) to β-actin. For A and C, data are presented as mean ± SD and one-way ANOVA followed by post hoc comparison revealed significant differences in promotion of sAPPα and inhibition of Aβ productions between OG versus EGCG (***P<0.001 with n = 3 for each condition).

In order to further test this hypothesis, N2a/APPwt cells were treated with OG or EGCG at 10 µM for 12 h and then cell lysates and conditioned media were prepared for determination of APP cleavage profiles using ELISA and IB analyses. In concert with reduced Aβ generation, EGCG and OG markedly increased sAPPα levels in conditioned media and increased α-CTF levels and ADAM10 maturation in cell lysates (Fig. 2A–E), a profile reflecting enhanced APP α-proteolysis. Taken together, these results confirm that APP α-proteolysis plays a role in the anti-amyloidogenic effect of EGCG and OG. Furthermore, OG activates these anti-amyloidogenic processes more effectively than EGCG. The phenolic compounds lacking the gallate moiety (C and EC) had little effect on sAPPα levels, ADAM10 maturation and amyloidogenesis (data not shown), suggesting that the gallate moiety may be an important functional component mediating promotion of APP α-secretase cleavage and anti-amyloidogenic effect of these compounds.

### ERα/PI3K/Akt Signaling is Involved in OG-mediated Promotion of APP α-proteolysis

Since our previous studies showed that EGCG promotes APP α-proteolysis and ADAM10 maturation *via* an ERα/PI3K/Akt dependent mechanism [18], we hypothesized that OG-elicited promotion of anti-amyloidogenic processing is also mediated by this mechanism. Initially, in order to explore the possibility of direct activation of ADAM10 by these compounds, N2a/APPwt broken cell preparations were generated. They were then treated with OG, AN, or EGCG at 10 µM for 1 h followed by ADAM10 analysis by IB. Densitometric analysis indicated that the ratios of mADAM10 to pADAM10 did not vary significantly with these treatments, indicating that ADAM10 activation by these compounds was not mediated through a direct molecular interaction but possibly involved in a signal transduction pathway (data not shown).

**Figure 3:**
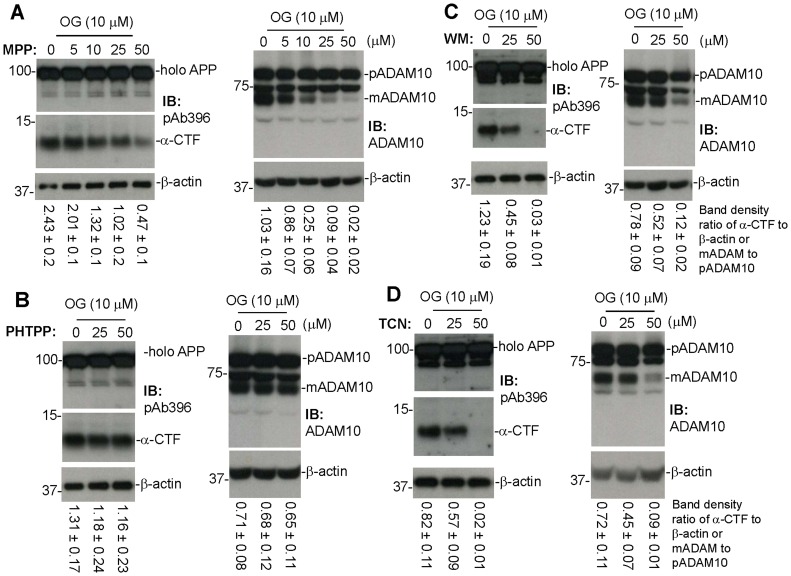
Estrogen receptor (ER) may mediate OG-promoted α-secretase cleavage of APP. Cultured N2a/APPwt cells were treated with OG at the most effective dose (10 µM) in presence of an ERα selective antagonist (MPP dihydrochloride) or an ERβ selective inhibitor (PHTPP) at various doses as indicated for 12 hours. Cell lysates were then prepared from these cells and subjected to IB analysis for APP processing into α-CTF (A and B, left) using an APP-carboxyl-terminal antibody (pAb396) and ADAM10 activation (A, B, right) using an anti-carboxyl-terminal ADAM10 antibody (ADAM10). Most notably, co-treatment of cultured N2a/APPwt cells with MPP (A), but not PHTPP (B), significantly suppresses OG’s promotion of ADAM10 activation and α-CTF cleavage. In addition, cultured N2a/APPwt cells were also treated with OG at 10 µM in presence of PI3K (wortmannin, WM) or Akt (TCN) inhibitor at various doses as indicated for 12 hours. Cell lysates were prepared from these treated cells and subjected to IB analysis for APP processing into α-CTF (C and D, left) and ADAM10 maturation (C and D, right). Both PI3K (WM) (C) and Akt (TCN) (D) inhibitors markedly reduced OG’s promotion of ADAM10 maturation and α-CTF cleavage. As shown below each IB panel, densitometry analysis shows the band density ratios (mean ± SD) of α-CTF to β-actin and mature ADAM10 (mADAM10) to pre-mature ADAM10 (pADAM10).

To determine the role of ERs in OG-mediated ADAM10 activation and APP α-proteolysis, cultured N2a/APPwt cells were treated with OG at 10 µM in the presence of the ERα selective antagonist, MPP dihydrochloride at 0, 5, 10, 25, and 50 µM for 12 hours, or the ERβ selective antagonist, PHTPP, at 0, 25, and 50 µM for 12 hours. Cell lysates were then prepared from these cells for determination of ADAM 10 maturation and the APP α-proteolytic product, α-CTF, by IB analyses. Co-treatment of cultured N2a/APPwt cells with MPP significantly suppressed OG-mediated promotion of α-CTF production and ADAM10 maturation (Fig. 3A). However, these effects were not observed after co-treatment with PHTPP (Fig. 3B). In addition, co-treatment of these cells with a PI3k antagonist, wortmannin (WM), or an Akt antagonist, TCN, at 0, 25, and 50 µM for 12 hours also significantly suppressed OG-mediated promotion of α-CTF production and ADAM10 maturation (Fig. 3C, D). These results indicate that OG-mediated promotion of APP α-proteolysis and ADAM10 maturation is indeed mediated by the ERα/PI3k/Akt signaling mechanism.

**Figure 4:**
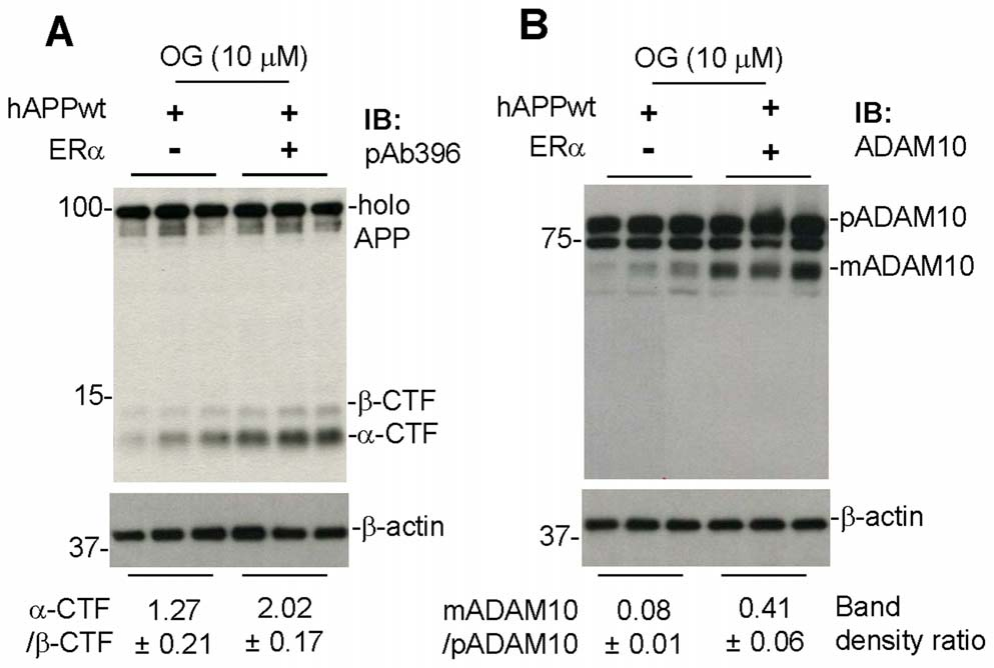
OG fails to promote α-secretase cleavage of APP in estrogen receptor α (ERα) deficient cells. We cultured primary cortical neuronal cells from brain tissues of one-day-old ERα deficient and intact mice. One week after the primary culture, these neuronal cells were transiently transfected with human wild-type APP_695_ plasmid DNA (hAPPwt) with Lipofectiamine™ LTX Reagent (as detailed in the method section). Twenty four hours later, these neuronal cells were treated with OG at 10 µM for 12 hours. Cell lysates were then prepared and subjected to IB analyses for α- and β-CTF (A) using pAb396 and ADAM10 maturation (B) using an anti-carboxyl-terminal ADAM10 antibody (ADAM10). Most notably, OG’s promotion of anti-amyloidogenic APP processing was significantly attenuated in the ERα null mouse-derived primary neuronal cells overexpressing hAPPwt. Human APP expression was examined after transient transfection by IB analysis using an anti-Aβ_1–17_ antibody (6E10) (data not shown). As shown below each IB, densitometry analysis shows the band density ratio (mean ± SD) of α-CTF to β-CTF and mature ADAM10 (mADAM10) to pre-mature ADAM10 (pADAM10).

Additional studies using primary cortical neuronal cells confirmed the role of ERα in OG-mediated promotion of APP α-proteolysis and ADAM10 maturation. Primary cortical neuronal cells were isolated from brain tissues of one-day-old ERα deficient or intact mice, cultured for 1 week and transiently transfected with human wild-type APP. Twenty four hours later, the cells were treated with OG at 10 µM for 12 h and then cell lysates were prepared and subjected to IB analyses of α-CTF production and ADAM10 maturation. OG-mediated α-CTF production and ADAM10 maturation were significantly attenuated in the ERα deficient neuronal cells overexpressing APP compared with the corresponding ERα intact neuronal cells, confirming the role of ERα in OG-mediated promotion of APP α-proteolysis and ADAM10 maturation (Fig. 4A, B).

### OG Markedly Increases APP α-proteolysis and Reduces Cerebral Amyloidosis in Tg2576 Mice

Having shown that OG markedly reduces Aβ generation in N2a/APPsw and N2a/APPwt cells together with enhanced APP α-proteolysis and ADAM10 maturation, we then determined if OG treatment could promote these anti-amyloidogenic processes and thereby impact Aβ levels in a transgenic Alzheimer’s mouse model. Beginning at 6 months of age, OG or EGCG (10 µg in 5 µl PBS, 20 mg/kg based on our previous study [14]) were i.c.v. injected daily for 1 week into Tg2576 mice. The mice were then sacrificed 24 h after the last injection and brain homogenates were prepared for determination of α-CTF, sAPPα, and Aβ_40_ and Aβ_42_ productions as well as ADAM10 maturation by IB analyses and/or ELISA.

**Figure 5:**
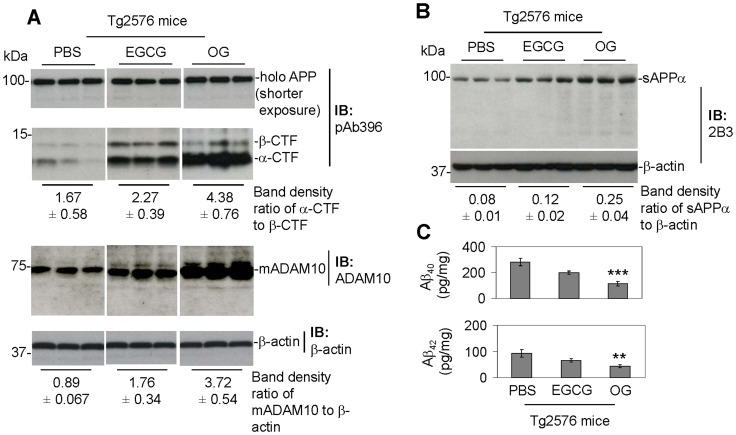
Tg2576 mice treated with OG showed markedly increased α-secretase cleavage of APP and significantly decreased levels of Aβ generation. A total of 13 Tg2576 female mice were used in this study over a period of one week. Five mice received OG or EGCG, and the remaining three received PBS. Beginning at 6 months of age (adult), Tg2576 mice were intracerebroventricular (i.c.v.) injected with OG or EGCG (10 µg in 5 µl PBS; this dose was suggested by our preliminary rangefinder study) or PBS (5 µl) daily through the implanted cannulae. Mice were sacrificed 24 h after the last injection for analysis of sAPPα, CTFs of APP, Aβs, and ADAM10 activation in brain homogenates using IB analyses and ELISA. IB analysis results show (A, top) holo APP and two bands corresponding to β-CTF and α-CTF by an APP-carboxyl-terminal antibody (pAb396), (A, bottom) mature ADAM10 (mADAM10) by an anti-carboxyl-terminal ADAM10 antibody (ADAM10) and (B) sAPPα production by a specific sAPPα antibody (2B3). As shown below each IB, densitometry analysis shows the band density ratios (mean ± SD) of α-CTF to β-CTF, mature ADAM10 (mADAM10) to β-actin and sAPPα to β-actin. (C) Detergent-soluble Aβs were analyzed by ELISA. Data are represented as mean ± SD of Aβ (pg/mg protein). For (C), a *t* test revealed a significant difference between OG- and EGCG-treated or PBS-injected Tg2576 mice for soluble total Aβ (Mean ± SD; **P<0.005; ***P<0.001). Most notably, OG-treated Tg2576 mice show much higher levels of APP processing into sAPPα and α-CTF as well as mADAM10 compared with either PBS- or EGCG-treated mice.

**Figure 6:**
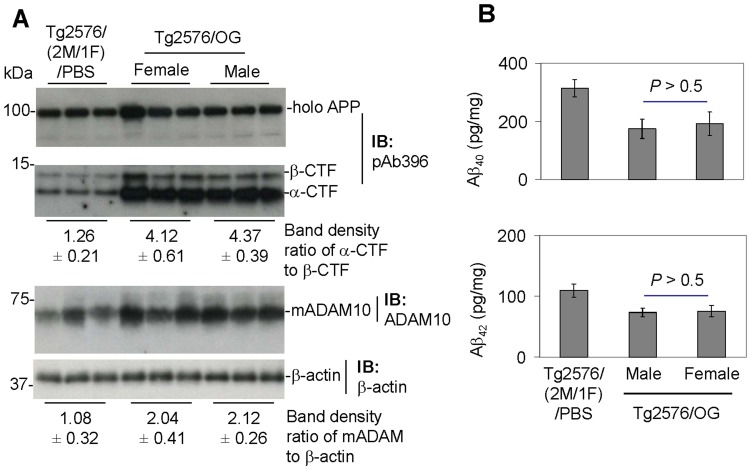
Female and male Tg2576 mice do not show a significant difference in terms ofα-secretase processing of APP and soluble cerebral Aβ levels upon OG treatment. A total of 13 Tg2576 mice were used in this study over a period of one week. Five female and five male Tg2576 mice received OG, and the remaining three mice (2 male/1 female) received PBS. Beginning at 6 months of age, these Tg2576 mice were i.c.v. injected with OG (10 µg in 5 µl PBS) or PBS (5 µl) daily through the implanted cannulae for one week. Mice were sacrificed 24 hours after the last injection for analysis of CTFs of APP, ADAM10 activation and Aβs in brain homogenates using IB analyses and ELISA. IB analysis show (A, top) holo APP and two bands corresponding to β-CTF and α-CTF by an APP-carboxyl-terminal antibody (pAb396) and (A, bottom) mADAM10 by an anti-carboxyl-terminal ADAM10 antibody (ADAM10). As shown below each IB, densitometry analysis shows the band density ratios (mean ± SD) of α-CTF to β-CTF and mADAM10 to β-actin. (B) Detergent-soluble Aβs were analyzed by ELISA. Data are represented as mean ± SD of Aβ (pg/mg protein). For (B), a *t*-test revealed no significant difference between female and male OG-treated Tg2576 mice for total soluble Aβ (*P*>0.05). Most notably, both OG-treated female and male Tg2576 mice show much higher levels of APP processing into α-CTF as well as mADAM10 compared with PBS-injected mice.

OG-treated mice showed much higher levels of α-CTF and sAPPα production and ADAM10 maturation, as well as lower levels of soluble brain Aβ, compared with either PBS- or EGCG-treated mice (Fig. 5A–C). In addition, OG-mediated α-CTF production, Aβ reduction, and ADAM10 maturation did not differ significantly between male and female Tg2576 mice (Fig. 6A, B). These results indicate that OG promotes anti-amyloidogenic APP α-processing and reduces cerebral amyloidosis in a mouse model of AD and that these anti-amyloidogenic effects of OG are elicited equally regardless of gender.

## Discussion

Over the past decade, intense focus has been given to the processes of APP and Aβ metabolism as possible therapeutic targets for AD [9]. APP is known to be metabolized by two pathways. The amyloidogenic pathway involves cleavage by β- and γ-secretases and, subsequently, the intracellular generation of Aβ [5]–[7]. Conversely, in the anti-amyloidogenic pathway, APP is cleaved by α-secretase within the Aβ domain, releasing sAPPα into the extracellular fluid and a carboxyl-terminal peptide, α-CTF, within the cell [23]. Since the α-secretase cleavage site is within the Aβ domain, α-secretase processing may preclude Aβ formation.

Many synthetic and naturally-occurring compounds have been investigated for their efficacy in modulating these processes. One such compound which has received worldwide attention for its therapeutic application in AD is found in green tea. In recent studies, EGCG, a polyphenolic flavonoid found in green tea, increased sAPPα secretion *via* activation of protein kinase C in SH-SY5Y neuroblastoma and PC12 pheochromocytoma cells [12], [13]. In concert with these findings, EGCG reduced Aβ generation together with increased ADAM10 activation, the putative α-secretase, and sAPPα and α-CTF production in N2a/APPsw cells [14], [15]. These studies indicate that EGCG mediates an anti-amyloidogenic effect presumably by activation of the APP α-processing pathway.

Several studies have shown that ERs are particularly important for cellular binding of several plant-derived polyphenolic compounds such as EGCG [16]. In the case of EGCG, its gallate group may correspond to the 7α position of estrogen, thereby facilitating ER binding [17]. Additional studies indicate that the ability of EGCG to increase APP α-processing is mediated by the ERα/PI3K/Akt signaling mechanism [18]. Several other naturally-occurring phenolic compounds, such as OG and AN, also contain the gallate group and potentially activate this mechanism. In addition, these gallate-containing phenolic compounds may have therapeutic efficacy in treating AD by activating ERα/PI3K/Akt signaling and thereby increasing anti-amyloidogenic APP α-processing.

In order to test this hypothesis, the present study investigated the efficacy of several gallate-containing phenolic compounds in reducing Aβ generation and promotion of sAPPα production in N2a/APPwt cells. Surprisingly, the results show that OG has greater efficacy compared with AN and EGCG in reducing Aβ generation and increasing sAPPα production (Fig. 1A–D). Other phenolic gallate-containing compounds were ineffective in reducing Aβ generation in these cells. Of particular interest, two compounds which lacked the gallate moiety, EC and C, also lacked this anti-amyloidogenic effect. Therefore, the gallate moiety may be an important functional component for eliciting this effect (Table 1). Similar to previous studies using EGCG [14], [15], the ability of OG to elicit this anti-amyloidogenic effect corresponded with its ability to activate ADAM10 (Fig. 2E) as well as increase sAPPα secretion and α-CTF production in these cells (Fig. 2A–E). Therefore, the anti-amyloidogenic effects of OG appear to be mediated by enhanced APP α-processing. Furthermore, these studies indicate that the Swedish mutation of APP is not necessary for these compounds to elicit their anti-amyloidogenic effect since similar effects of these compounds were observed in APP-overexpressing cells lacking this mutation.

In addition, the present study shows that the anti-amyloidogenic effects of OG are mediated by activation of the ERα/PI3K/Akt signaling mechanism (Fig. 3A, C–D), as shown previously for EGCG [18]. In particular, the ability of OG to promote ADAM10 maturation and increase α-CTF formation in N2a/APPwt cells was reduced by specific inhibitors of ERα, PI3K, and Akt. Furthermore, OG-mediated ADAM10 maturation and α-CTF formation were reduced in primary cortical neurons from ERα deficient mice (Fig. 4A, B), confirming the role of ERα in these effects. In contrast, the present study showed that EGCG, OG, and AN were unable to activate ADAM10 in broken cell preparations (data not shown), indicating that these compounds cannot activate this secretase directly but require a signaling mechanism. In addition, the ability of OG to activate ADAM10 and increase α-CTF production was unaffected by PHTPP, an inhibitor of ERβ, indicating that the anti-amyloidogenic effect of this compound is not mediated by the ERβ subtype (Fig. 3B).

Previous studies indicated that EGCG-mediated neuroprotection may also involve mitogen activated protein kinase/extracellular signal-regulated kinase (MAPK/ERK) signaling [24], [25] and that EGCG can enhance the ADAM10 activating enzymes PC7 and furin independent of PI3K activation in N2a cells and others [18]. Future studies should therefore investigate the role of these other signaling mechanisms in the anti-amyloidogenic effects of EGCG and OG.

Of particular importance is the finding that OG markedly reduced cerebral amyloidosis, together with enhanced α-CTF and sAPPα production as well as ADAM10 maturation, in a mouse model of AD (Tg2576 mice; Fig. 5A–C, 6A, B). This is in accord with others demonstrating a moderate neuronal overexpression of ADAM10 in mice transgenic for human APP increased the secretion of the neurotrophic sAPPα, reduced the formation of Aβs, and prevented their deposition in plaque [26]. As clinical support, sAPPα is reduced in the cerebrospinal fluid of AD patients [27], [28], indirectly suggesting that anti-amyloidogenic (α-secretase) APP processing is impaired in AD. Since α-secretase cleaves within the Aβ peptide domain, its activation may even have the added advantage of not only generating the putatively neuroprotective sAPPα [29], but also precluding neurotoxic Aβ peptide formation.

Furthermore, the present study indicates that OG is equally effective in eliciting these anti-amyloidogenic effects in both male and female mice (Fig. 6A, B), indicating that activation of α-secretase can be an equally effective treatment for AD in both male and female individuals. This finding is particularly important in light of studies showing that postmenopausal women may be particularly vulnerable to developing AD [30], [31] and that this increased risk can be partially attributed to estrogen depletion [31]. Further studies should be performed to determine the efficacy of OG in slowing or reversing the cognitive and behavioral impairment which develop in mouse and other models of AD, particularly that associated with the female gender.

In conclusion, the present study indicates that two naturally-occurring phenolic compounds, namely OG and AN, have greater efficacy than EGCG in reducing Aβ generation in neuronal and mouse models of AD. The anti-amyloidogenic effect of these compounds appears to be mediated by enhanced APP α-processing and ADAM10 maturation. In addition, the present study shows that the anti-amyloidogenic effect of these compounds is mediated by activation of the ERα/PI3K/Akt signaling mechanism. Given that toxic oligomeric species of Aβ may play an important role in the development of AD and other neurodegenerative disorders, further exploration of the use of naturally-occurring phenolic compounds, in particular OG, in the prevention or treatment of these disorders is warranted.
